# Cascade Signals of Papaverine Inhibiting LPS-Induced Retinal Microglial Activation

**DOI:** 10.1007/s12031-019-01289-w

**Published:** 2019-03-09

**Authors:** Ting Zhou, Yu Zhu

**Affiliations:** Department of Ophthalmology, The First Affiliated Hospital of Zhengzhou University, East Jianshe Road, Zhengzhou, 450001 People’s Republic of China

**Keywords:** Papaverine, Primary microglia, MEK/Erk, cAMP/PKA

## Abstract

Studies have shown that papaverine can inhibit lipopolysaccharide (LPS)-induced microglial activation. The retinal primary microglia of newborn SD rats were isolated and purified, and a LPS-induced microglia activation model was established. The protein phosphorylation level of the signaling pathway was detected by western blotting. The transcription and expression of TNF-α, IL-1β, and IL-10 were respectively detected by RT-PCR and ELISA to observe the abnormal activation of primary microglia. The cAMP inhibitor Rp-isomer, PKA inhibitor H89, and MEK inhibitor U0126 were separately added to further investigate the role of MEK/Erk in PAP inhibition of primary microglial activation and the relationship between cAMP/PKA and MEK/Erk. It was found that the level of MEK phosphorylation was upregulated after LPS stimulation, which was blocked by 10 μg/ml of papaverine.10μM U0126 significantly inhibited TNF-α and IL-1β and increased IL-10 transcription and expression in retinal microglia (*P* < 0.01). Both Rp-isomer and H89 upregulated the phosphorylation levels of MEK and Erk. Papaverine may inhibit inflammatory factors and promote the expression of anti-inflammatory factors through the cAMP/PKA and MEK/Erk pathway, thereby inhibiting LPS-induced activation of primary retinal microglia, and the MEK/Erk pathway may be partially regulated by cAMP/PKA, which can provide theoretical basis and experimental basis for its protection of the central nervous system.

## Introduction

Papaverine is a non-selective PDE inhibitor that relaxes cardiovascular, respiratory, and gastrointestinal smooth muscles and is commonly used in cerebral thrombosis, pulmonary embolism, and arterial spasm (Zhu et al. 2014; Kim et al. 2014). In recent years, its role in inhibiting microglia immune inflammation and central nervous system protection has gradually received attention. Yoshikawa et al. first found that papaverine could inhibit release of TNF-α and IL-1β in LPS-induced BV2 cells (Yoshikawa et al. 1999). Our study also found that papaverine inhibited the activation of primary retinal microglia by inhibiting the expression of inflammatory factors and promoting the expression of anti-inflammatory factors.

Activation of microglia is a double-edged sword (Loane and Kumar 2016), and functional changes in activated microglia mainly include enhanced phagocytic capacity and release of inflammatory mediators (Kettenmann et al. 2011). In the early stage of central nervous system inflammation, activated microglia can limit the spread of inflammation, phagocytose pathogens, and play a neuroprotective role (Namiki et al. 2000; Iannotti et al. 2004), which helps maintain central nervous system homeostasis (Kettenmann et al. 2011). The downside is that chronic abnormally activated microglia release inflammatory mediators and excessively phagocytose injured neurons. Recent studies have shown that abnormally activated microglia can phagocytose active neurons under stress, which called phagocytic apoptosis (Brown and Neher 2014). Inflammatory mediators play a central role in neurodegenerative diseases (Alam et al. 2016), while microglia are the major cells that release inflammatory mediators (Buttini et al. 1997; Ghosh et al. 2012). TNF-α and IL-1β are cytotoxic inflammatory mediators released by microglia in early retinal injury, which initiate a series of cellular responses, including activation and migration of microglia and astrocytes (Pousset et al. 1999; Milner and Campbell 2002; Tanuma et al. 2006). Some studies had shown that inhibition of TNF-α and IL-1β could significantly reduce ganglion cell apoptosis (Sivakumar et al. 2011). In some cases, inhibition of activated microglia could increase the release of anti-inflammatory factors, such as IL-10 (Yoshikawa et al. 1999; Mizuno et al. 2004; Woo et al. 2004; Schafer 2012) to protect central nervous cells and promote axonal regeneration. Mizuno et al. found that Ibudialast can increase the expression of IL-10, NGF, and GDNF and inhibit neuronal apoptosis in a dose-dependent way (Mizuno et al. 2004).

Our preliminary experiments had proposed that papaverine inhibited the release of inflammatory cytokines in primary retinal microglia. However, further mechanisms remain unclear. Dang et al. found that tetrandrine could inhibit the activation of BV2 cells by inhibiting NF-kB and ERK signaling pathways (Dang et al. 2014). Mitogen-activated protein kinases (MAPKs) are essential signaling molecules involved in the control of a variety of macrophage mediators. Studies have shown that MAPKs are involved in microglial activation and appear to play crucial roles in the inflammatory process (Kim et al. 2004; Zhang and Dong 2007). To further investigate the mechanism of papaverine inhibition of microglial activation, we investigated the involvement of MEK/Erk during the activation of microglia and analyzed the relationship between cAMP/PKA and MEK/Erk signaling pathway, which could explore the mechanism of papaverine regulating the functional status of retinal microglia and provide theoretical basis and experimental basis for its protection of the central nervous system.

## Methods

### Cell Culture and Treatment

Retina primary microglia were isolated from SD rats provided by the Experimental Animal Center of Zhengzhou University. Cells were cultured in medium containing DMEM/F12 (Hyclone, GE Healthcare),10% fetal bovine serum (FBS), and 1% penicillin/streptomycin. Cells were pretreated with cAMP inhibitor Rp-isomer(200 μmol,C0735-1VL,Sigma, USA), PKA inhibitor H89(5 μmol, 9844,CST) and Erk inhibitor U0126 (10 μmol, HY-12031,MCE) for 1 h, papaverine (10 μg/ml,120,901–1, Northeast Pharmaceutical Group Co. Ltd., China) for 4 h, and incubated with LPS (100 ng/ml) for 24 h. All animals used for cell culture were approved by the institutional committee of the Animal Research Committee and the Animal Ethics Committee of Zhengzhou University.

### Western Blotting

To extract total cell protein, cells were centrifuged and collected, and washed three times with PBS for 3 min. One hundred microliters of RIPA lysate was added and calls were lysed on ice for half an hour. The extracted protein was detected using the BCA method. Absorbance value was measured by the microplate reader; The protein concentrations of samples were calculated from the standard curve. Samples were analyzed on 10% SDS-PAGE gels and transferred to NC membranes. Then the NC membrane was placed into a blocking solution (5% skim milk powder dissolved in TBST) for 1 h. Primary antibodies were added and incubated overnight at 4 °C. On the next day, after secondary antibodies added, samples were incubated in the dark for 1–2 h at room temperature. The NC membrane was infiltrated with the mixed chemiluminescence reagent. Images were analyzed and the gray value was analyzed by Image J software. The specific primary antibodies included Phospho-MEK1/2 (#9154, Cell Signaling, USA), Phospho-ERK1/2 (214362, Abcam), and β-actin (ab3280, Abcam). Experiments were repeated at more than three times.

### Reverse Transcription PCR

Total RNA was extracted using Trizol reagent according to the instructions provided by the suppliers, and the RNA concentration was measured using a nucleic acid micro spectrophotometer. RNA was reverse-transcribed using a HiScript® II Q RT SuperMix kit(R223-01, Vazyme, China). Then, cDNA was amplified by PCR. The reaction conditions for amplifying DNA were 95 °C for 10 min, followed by 40 cycles, each cycle consisting of 95 °C for 10 s and 60 °C for 30 s, and testing with the ABI 7500Fast. The mRNA expression was normalized to the expression level of GAPDH. The sequence-specific oligonucleotide primers were as follows: TNF-α forward: AGACCCTCACACTCAGATCA, reverse: GTCTTTGAGATCCATGCCATTG; IL-1β forward: CACCTCTCAAGCAGAGCACAG, reverse: GGGTTCCATGGTGAAGTCAAC; IL-10 forward: GCCAAGCCTTGTCAGAAATGA, reverse: TTTCTGGGGCCATGGTTCTCT; GAPDH forward: TGCACCACCAACTGCTTAGC, reverse: GCCCCACGGCCATCA. The results were calculated using the 2^-△△ct^ method, and statistical analysis was performed. Experiments were repeated at more than three times.

### Enzyme-Linked Immunosorbent Assay

The culture mediums were collected and centrifuged to remove floating cells. The supernatant was used for ELISA assay. The expression of TNF-α, IL-1β, and IL-10 was measured using an ELISA kit in accordance with the protocol provided by the manufacturer. Briefly, different samples and the detection antibody were added to each well. After incubated for 30 min, the plates were washed, and aenzyme and chromogenic substrate were added. Then stop solution was added to terminate the reaction. Finally, the absorbance value at 450 nm was measured with a microplate reader to determine the concentration of each well. ELISA kits used were Rat cAMP ELISA kit (ab133039, Abcam), Rat TNF-α ELISA kit (12-3720, Dakewe, China), Rat IL-1β ELISA kit (12-3012, Dakewe, China), and Rat IL-10 ELISA kit(12-3100, Dakewe, China). Experiments were repeated at more than three times.

### Statistical analyses

Data were expressed as the mean ± SEM (standard error of the mean). Statistical significance of the differences between experimental groups was evaluated by one-way analysis of variance followed by Dunnett’s test. The differences between two groups were assessed using *t* test. Differences were considered statistically significant at *P* < 0.05.

## Results

### Effects of Papaverine on the Transcription and Expression of TNF-α, IL-1β, and IL-10

**Fig. 1:**
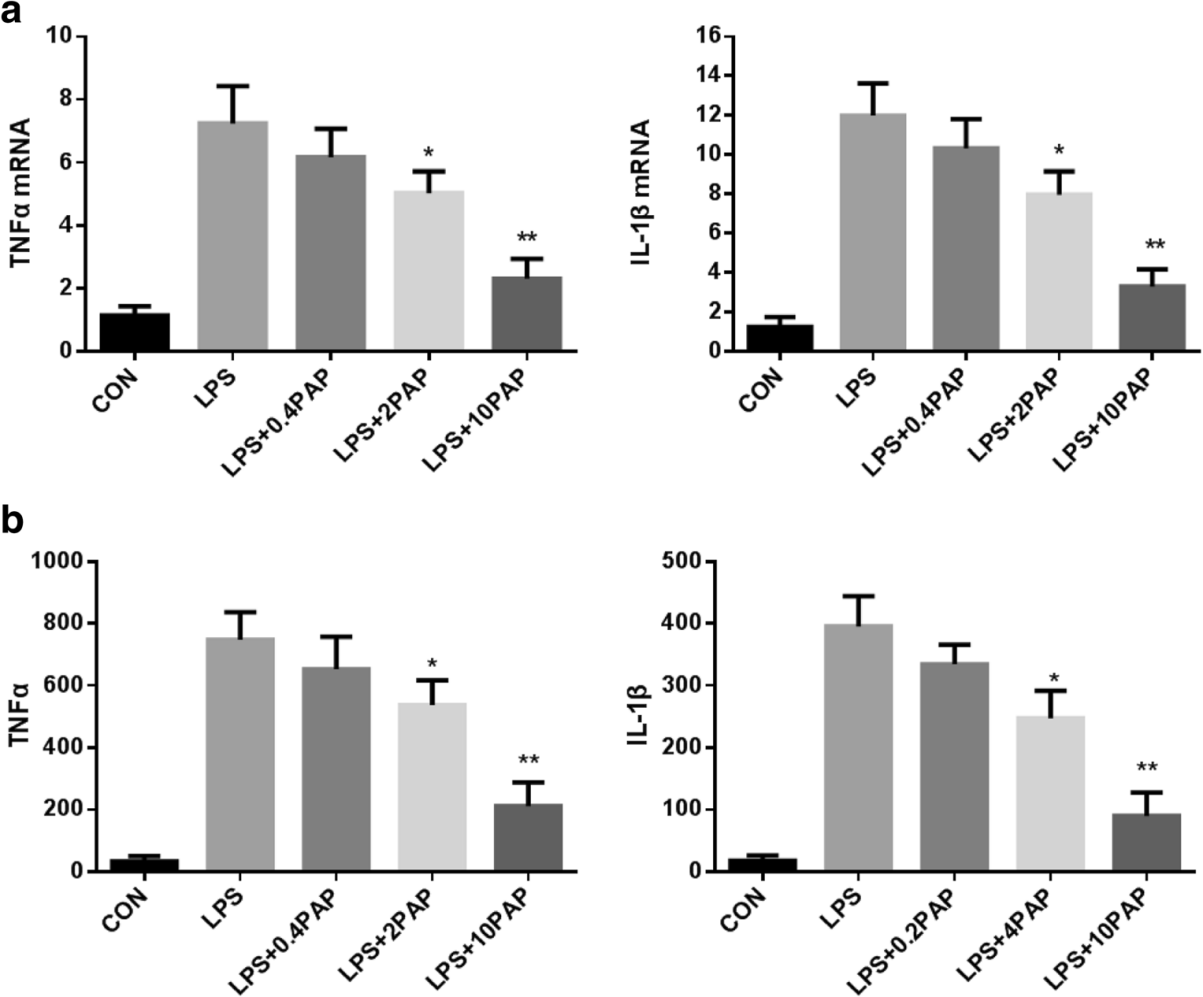
The transcription and expression of TNF-α and IL-1β after papaverine pretreatment. Primary microglia were pretreated with papaverine (0, 0.4, 2, and 10 μg/ml) for 4 h and incubated with LPS (100 ng/ml) for another 24 h. **a** The transcription of TNF-α and IL-1β were detected by RT-PCR (*n* = 5). **b** The expression of TNF-α and IL-1β were detected by ELISA (*n* = 5). *P < 0.05 versus LPS group, **P < 0.01 versus LPS group

Papaverine dose-dependently suppressed mRNA expression of TNF-α and IL-1β in LPS-stimulated microglia (*n* = 5; *P* < 0.05 compared with the LPS group, Fig.1a), and these results were consistent with changes in TNF-α and IL-1β expression (*n* = 5; *P* < 0.05 compared with the LPS group, Fig.1b).

**Fig. 2:**
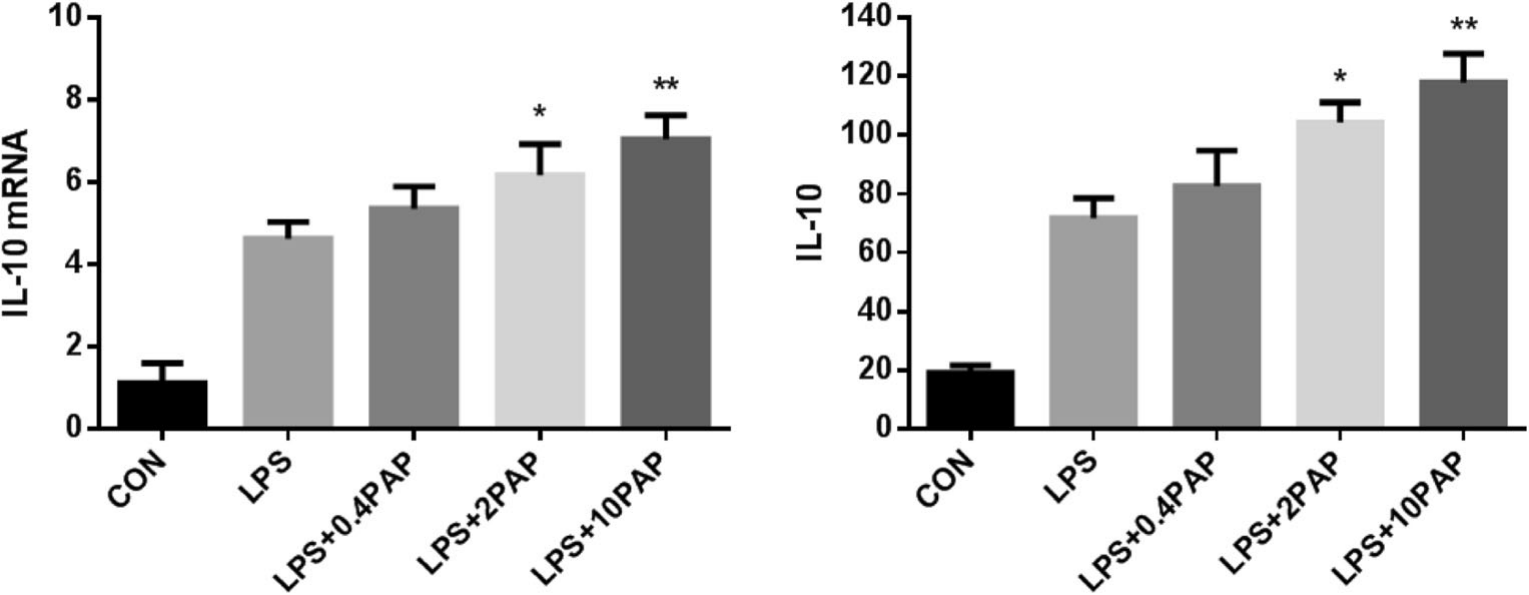
The transcription and expression of IL-10 after papaverine pretreatment. Primary microglia were pretreated with papaverine (0, 0.4, 2, and 10 μg/ml) for 4 h and stimulated by LPS for another 1 day. The transcription and expression of IL-10 were detected by RT-PCR and ELISA, respectively (*n* = 5). *P < 0.05 versus LPS group, **P < 0.01 versus LPS group

The addition of LPS enhanced the transcription and expression of IL-10, and pretreatment of PAP was able to further enhance these effects (*n* = 5; *P* < 0.05 compared with the LPS group, except for the 0.4 μg/ml group, Fig.2).

### Papaverine Suppresses TNF-α and IL-1β by Activating cAMP/PKA Signaling Pathway

**Fig. 3:**
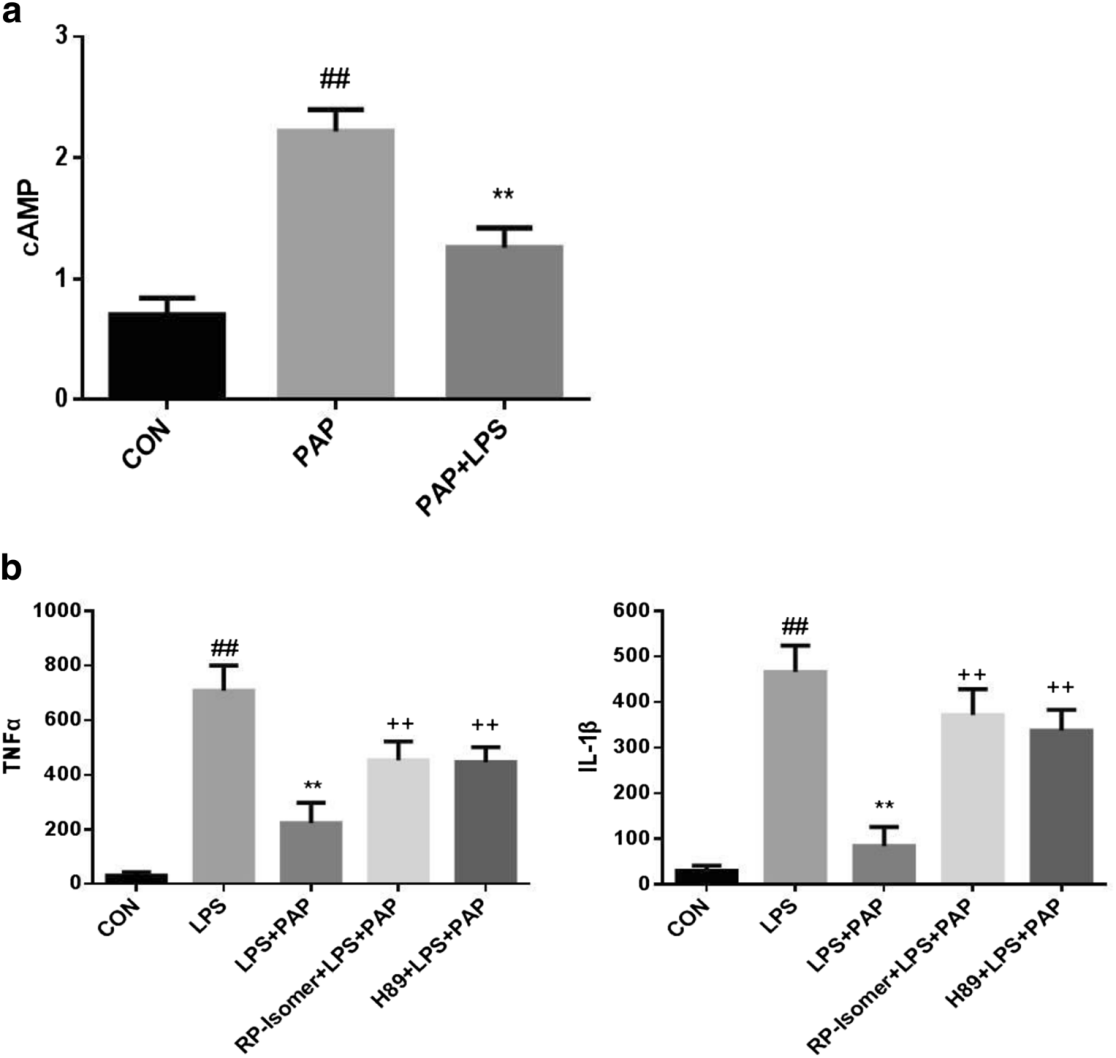
Papaverine suppresses TNF-α and IL-1β by cAMP/PKA signaling pathway. **a** Treatment with 10 μg/ml of papaverine significantly upregulates cAMP (*n* = 5, *P* < 0.01 compared with CON group), while LPS can partly inhibit the increase of cAMP (*n* = 5, *P* < 0.01 compared with PAP group; *P* < 0.05 compared with CON group). ##*P* < 0.01 versus CON group, **P < 0.01 versus PAP group. **b** Primary retinal microglia were pretreated with 200 μmol Rp-isomer and 5 μmol H89 for 30 min, then treated with 10 μg/ml of papaverine for 4 h, and finally incubated with 100 ng/ml LPS for 1 h. The expression level of TNF-α and IL-lβ were detected by ELISA. The results showed that papaverine could inhibit the expression of TNF-α and IL-1β which upregulated by LPS (*n* = 5). After adding Rp-isomer, the expression of TNF-α and IL-1β were increased (*n* = 5). Similarly, the expression of TNF-α and IL-1β were increased after adding H89 (*n* = 5). ##*P* < 0.01 versus CON group, **P < 0.01 versus LPS group, ++ *P* < 0.01 versus LPS + PAP group

The expression of cAMP was detected by ELISA. As shown in Fig.3a, primary retinal microglia were divided into three groups after digestion and adherence: (1) normal medium; (2) 10 μg/ml papaverine pretreated for 30 min; (3) 10 μg/ml papaverine pretreated for 30 min and incubated with LPS for 1 h. Treatment with 10 μg/ml of papaverine significantly upregulates cAMP(2.219 ± 0.0 90 pmol/ml; *n* = 5; *P* < 0.01 compared with the control group), while after 1 h of LPS treatment, cAMP is significantly decreased (1.256 ± 0.0 82 pmol/ml; *n* = 5; *P* < 0.01 compared with the PAP group).

Then, we determined whether activation of the cAMP/PKA pathway leads to inhibition of TNF-α and IL-lβ. We used the cAMP inhibitor Rp-isomer (200 μmol) and the PKA inhibitor H89 (5 μmol) to block the cAMP/PKA pathway. The expression level of TNF-α and IL-lβ were detected by ELISA. The results showed us that inhibition of the cAMP/PKA pathway could increase the release of TNF-α and IL-lβ. As shown in Fig.3b, we found that papaverine could significantly inhibit the expression of TNF-α and IL-1β which upregulated by LPS (*n* = 5; *P* < 0.01 compared with the LPS group). After adding Rp-isomer, the expression of TNF-α and IL-1β were increased (*n* = 5; *P* < 0.01 compared with the LPS+PAP group). Similarly, the expression of TNF-α and IL-1β were increased after adding H89 (*n* = 5; *P* < 0.01 compared with the LPS+PAP group).

### Papaverine Downregulates MEK/Erk Signaling Pathway

**Fig. 4:**
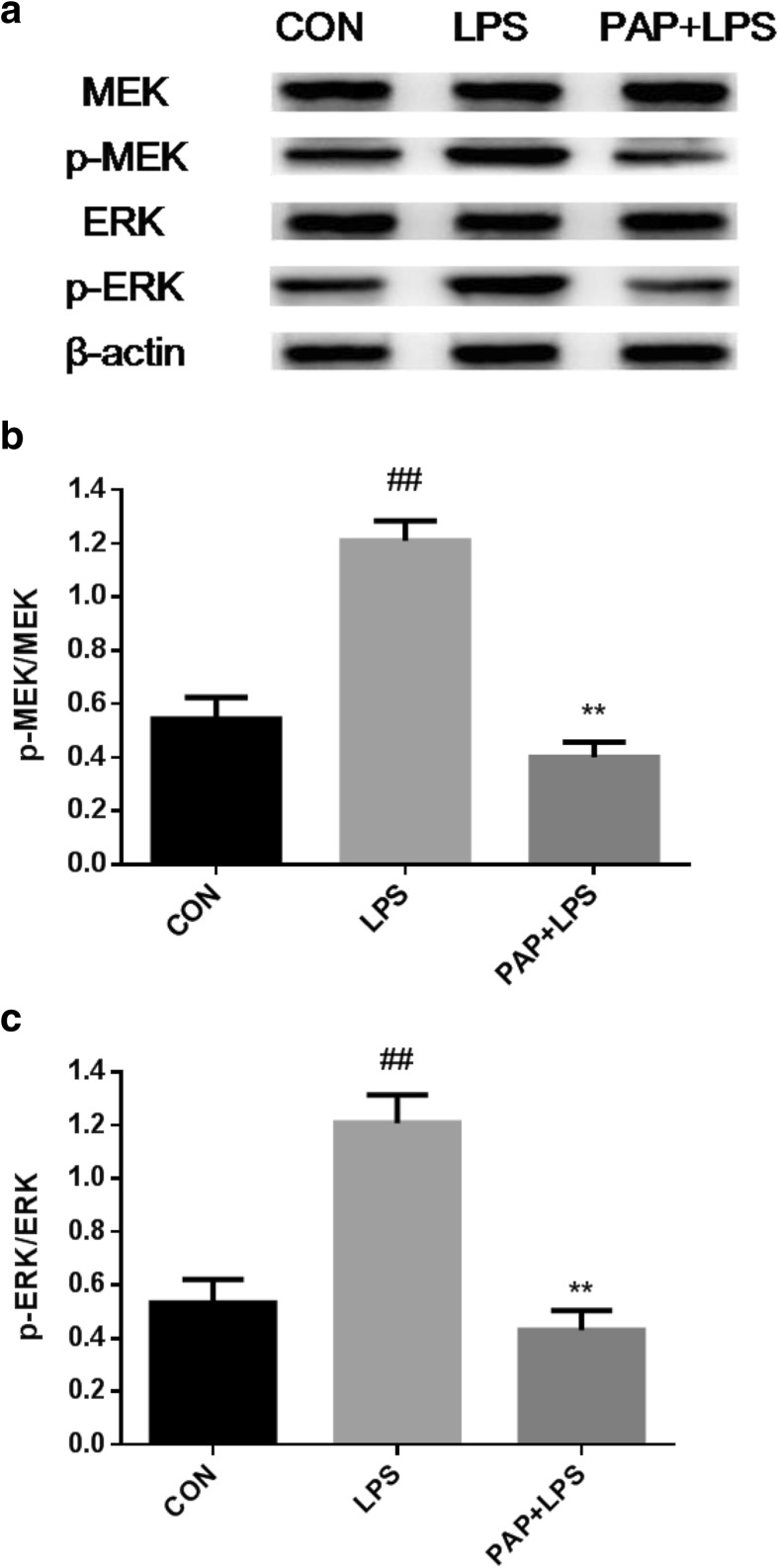
**a**–**c** Papaverine inhibited phosphorylation of MEK and Erk in LPS-induced microglia. Primary retinal microglia were pretreated with 10 μg/ml papaverine for 4 h, and 100 ng/ml of LPS was added for further 1 h. The cells were lysed, total protein was extracted, and the expression of phosphorylated MEK and Erk were detected by Western Blotting. The results showed that LPS significantly upregulated the phosphorylation levels of MEK and Erk, and papaverine blocked the above effects (*n* = 4). ##*P* < 0.01 versus CON group, **P < 0.01 versus LPS group

The phosphorylation levels of MEK and Erk were detected by Western Blotting. As shown in Fig. 4, the expression of p-MEK and p-Erk were upregulated after stimulation with LPS at 100 ng/ml for 1 h (1.21 ± 0.033, 1.209 ± 0.053, respectively; *n* = 4; *P* < 0.01 compared with the control group), and these effects were blocked by 10 μg/ml of papaverine (0.399 ± 0.026, 0.43 ± 0.037, respectively; *n* = 4; *P* < 0.01 compared with the LPS group).

### U0126 Attenuates the Transcription and Expression of Inflammatory Factors TNF-α and IL-1β

**Fig. 5:**
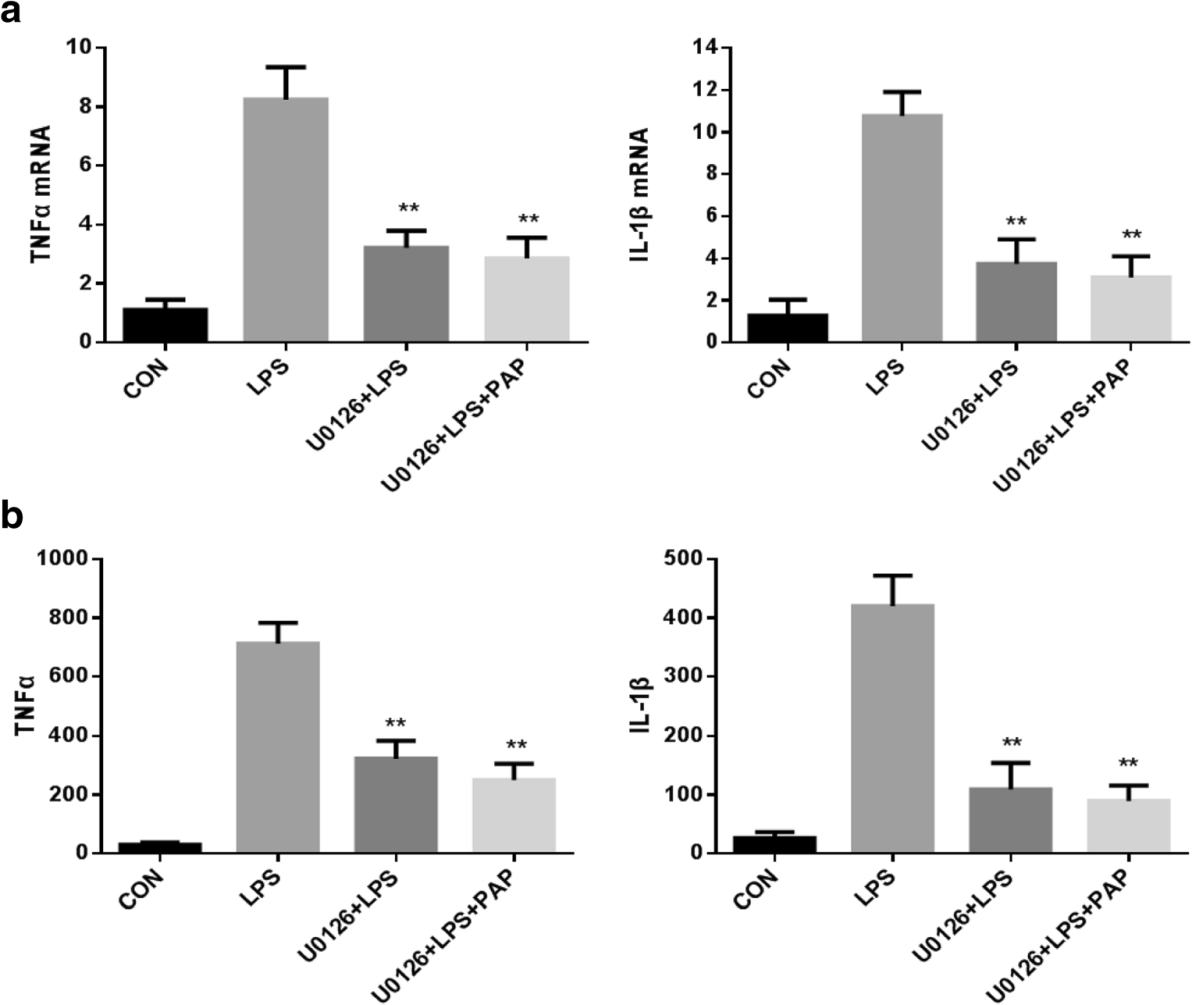
**a**, **b** U0126 inhibited the transcription and expression of TNF-α and IL-1β. Primary retinal microglia were pretreated with 10 μmol U0126 for 1 h, then treated with 10 μg/ml of papaverine for 4 h, and finally added with 100 ng/ml LPS for 1 h. The cells were harvested, RNA was extracted, the cDNA was synthesized, and the concentration of TNF-α mRNA and IL-1β mRNA were detected by RT-PCR. The supernatant was collected and the concentrations of TNF-α and IL-1β were detected by ELISA. The results showed that U0126 significantly inhibited the transcription (a) and expression (b) of TNF-α and IL-1β that upregulated by LPS (*n* = 5, *P* < 0.01 compared with the LPS group). **P < 0.01 versus LPS group

We next determined whether inhibition of the MEK/Erk pathway results in inhibition of microglial activation. The results showed us that activation of the MEK/Erk pathway could increase the release of inflammatory factors such as TNF-α and IL-lβ. As shown in Fig.5, the transcription level of inflammatory factors was detected by RT-PCR, and it was found that 100 ng/ml of LPS increased 7.4-fold TNF-α mRNA and 8.5-fold IL-lβ mRNA (*n* = 5; *P* < 0.01 compared with the control group, Fig.5a) compared with physiological state, while addition of 10 μM U0126 significantly inhibited retinal microglia 61.1% TNF-α mRNA and 65.4% IL-1β mRNA (*n* = 5; *P* < 0.01 compared with the LPS group, Fig.5a), the mRNA of TNF-α and IL-1β after using papaverine+U0126 were less than using U0126 alone, but it was not statistically significant (*n* = 5; *P* = 0.538, 0.505, respectively, compared with the U0126+LPS group; Fig.5a).

Consistent with the results of PT-PCR, the expression level of inflammatory factors was detected by ELISA, and found that the content of TNF-α and IL-1β was low under physiological conditions, while LPS upregulated 23.8 times TNF-α and 16.2 times IL-1β (*n* = 5; compared with the control group, *P* < 0.01, Fig.5b). The expression levels of TNF-α and IL-1β were significantly reduced after adding U0126 (*n* = 5; compared with the LPS group, *P* < 0.01, Fig.5b), and the expression of TNF-α and IL-1β in papaverine+U0126+LPS group was less than in U0126+LPS group, but it was not statistically significant (*n* = 5; *P* = 0.086, 0.424, respectively, compared with the U0126+LPS group; Fig.5b).

### U0126 Increases Transcription and Expression of the Anti-Inflammatory Factor IL-10

**Fig. 6:**
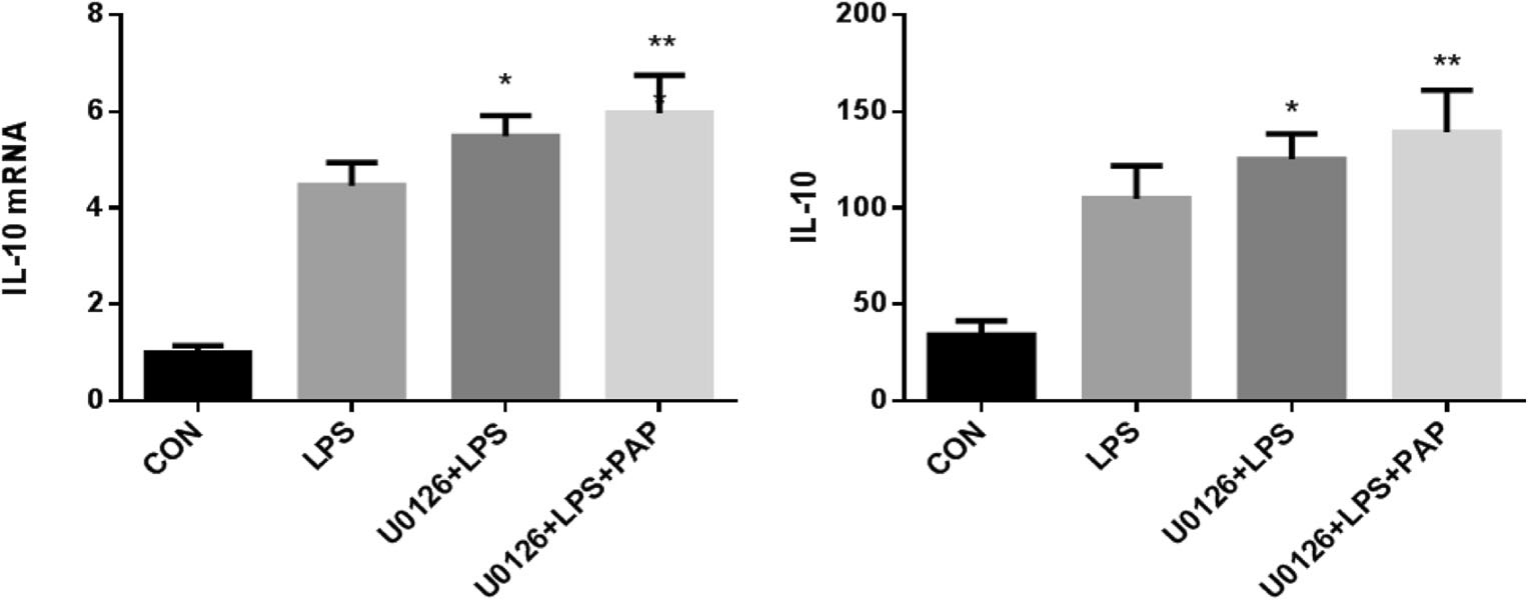
U0126 increased the transcription and expression of IL-10. Primary retinal microglia were pretreated with 10 μmol of U0126 for 1 h, then treated with 10 μg/ml of papaverine for 4 h, and finally added with 100 ng/ml of LPS for 1 h. Cells were harvested and RNA was extracted. The cDNA was synthesized and the concentration of IL-10 mRNA was detected by RT-PCR. The supernatant was collected and the concentration of IL-10 was detected by ELISA. The results showed that U0126 can further upregulate the transcription and expression of IL-10(*n* = 5, *P* < 0.01 compared with the LPS group). *P < 0.05 versus LPS group, **P < 0.01 versus LPS group

As shown in Fig.6, the transcription of IL-10 was detected by RT-PCR, and it was found that LPS increased IL-10 mRNA compared with physiological state and 10 μM U0126 further increase IL-10 mRNA(*n* = 5; compared with the LPS group, *P* < 0.05, Fig.6), and the mRNA of IL-10 after using papaverine+U0126 were higher than using U0126 alone, but it was not statistically significant (*n* = 5; *P* = 0.402 compared with the U0126+LPS group; Fig.6).

Consistent with the results of PT-PCR, the expression level of IL-10 was detected by ELISA, and found that a certain amount of IL-10 existed under physiological conditions, while LPS upregulated IL-10, addition of U0126 further increase IL-10(*n* = 5; compared with the LPS group, *P* < 0.05, Fig.6), and the expression of IL-10 in papaverine+U0126+LPS group were higher than in U0126+LPS group, but it was not statistically significant (*n* = 5; *P* = 0.252 compared with the U0126+LPS group; Fig.6).

### The Activation of the MEK/Erk Pathway Is Regulated by the cAMP/PKA Pathway

**Fig. 7:**
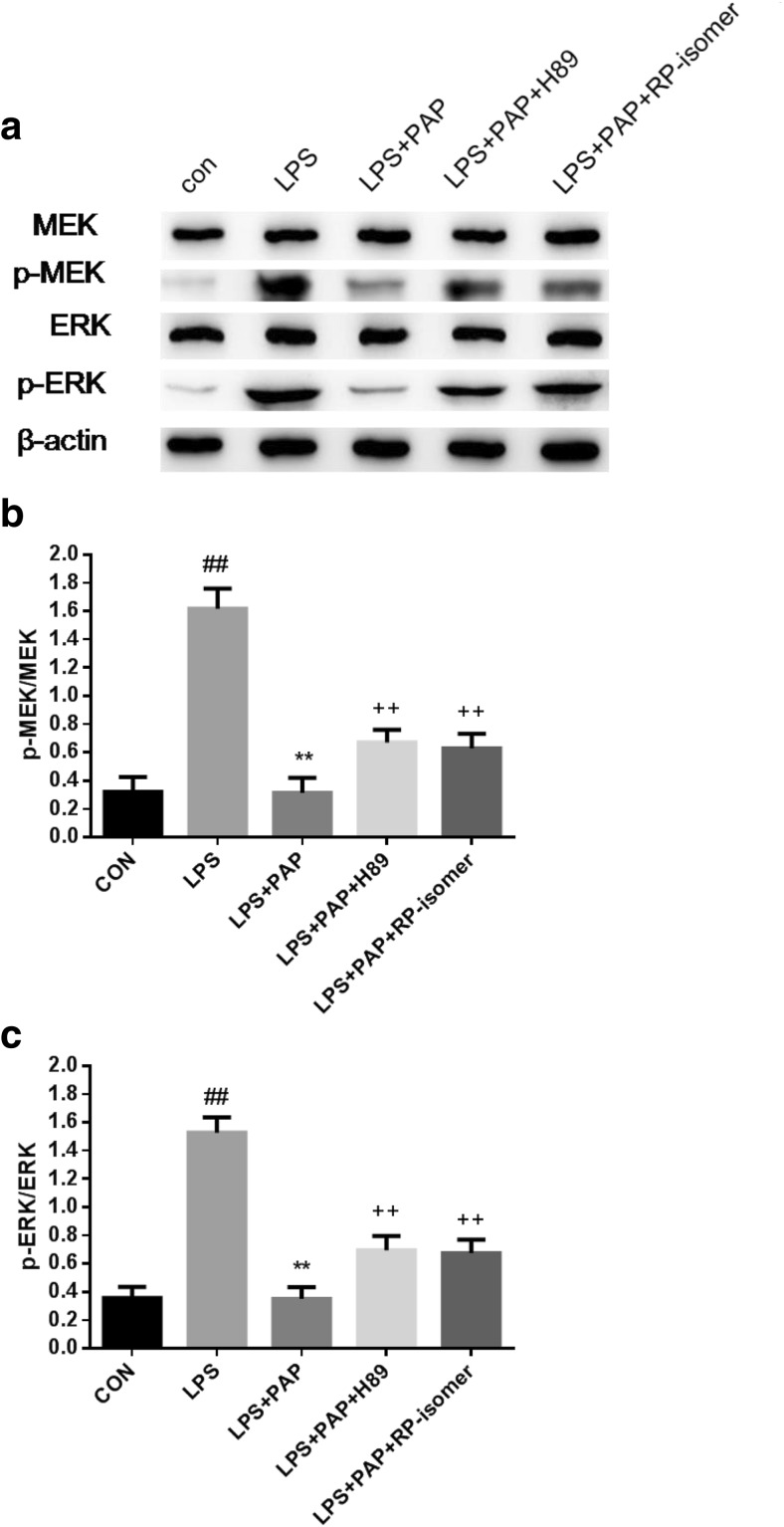
**a**–**c** H89 and Rp-isomer blocked the inhibition of papaverine on the phosphorylated MEK and Erk in LPS-induced microglia. Primary retinal microglia were pretreated with 200 μmol Rp-isomer and 5 μmol H89 for 30 min, then treated with 10 μg/ml of papaverine for 4 h, and finally incubated with 100 ng/ml LPS for 1 h. The cells were lysed, total protein was extracted, and the expression of phosphorylated MEK and Erk were detected by Western Blotting. The results showed that papaverine could block the phosphorylation of MEK and Erk that upregulated by LPS, while Rp-isomer and H89 partly blocked these effects caused by papaverine (*n* = 6). ##*P* < 0.01 versus CON group, **P < 0.01 versus LPS group, ++ *P* < 0.01 versus LPS+PAP group

In this study, we found that papaverine could activate the cAMP and inhibit the activation of the MEK/Erk pathway. However, the relationship between the two pathways is not well understood. Therefore, we added the cAMP inhibitor Rp-isomer (200 μmol) and the PKA inhibitor H89 (5 μmol) to block the cAMP/PKA pathway. The phosphorylation of MEK and Erk were detected by Western Blotting. As shown in Fig. 7, after 1 h of LPS stimulation, the phosphorylation levels of MEK and Erk were significantly upregulated (1.618 ± 0.071, 1.528 ± 0.054, respectively; *n* = 6; *P* < 0.01 compared with the control group), and papaverine reversed the above effects(0.314 ± 0.054, 0.35 ± 0.042, respectively; *n* = 6; *P* < 0.01 compared with the LPS group). Precipitating Rp-isomer (0.629 ± 0.052, 0.674 ± 0.048, respectively; *n* = 6; *P* < 0.01 compared with the LPS+PAP group) and H89 (0.67 ± 0.046, 694 ± 0.051, respectively; *n* = 6; *P* < 0.01 compared with the LPS+PAP group) for 30 min before the administration of papaverine significantly increased phosphorylated MEK and Erk and partly blocked the effects caused by papaverine.

## Discussion

Our studies indicated that papaverine dose-dependently inhibited the transcription and expression of the inflammatory cytokines TNF-α and IL-1β, promoted the expression of the anti-inflammatory factor IL-10 of microglia. The above effects were achieved by the cAMP/PKA and MEK/Erk signaling pathway. Then we further investigate the possible mechanism of papaverine and find that papaverine can inhibit the MEK/Erk pathway, thereby inhibiting microglial activation, and MEK/Erk may be regulated by cAMP/PKA. Our results have certain significance as papaverine has been approved by the Food and Drug Administration for the treatment of patients with cerebral thrombosis, pulmonary embolism, arterial spasm, etc. (Zhu et al. 2014; Kim et al. 2014), which is clinically safe. It has been detected through previous studies that conventional doses of papaverine can inhibit retinal microglial activation, which is clinically feasible. Besides, we discovered that papaverine can directly inhibit MEK/Erk pathway and regulate MEK/Erk through cAMP/PKA pathway. We speculate that, together with various upstream factors and downstream substrates, the mechanism of action of papaverine constitutes a functional and responsive signal transduction network.

It is known that after LPS induction, multiple signaling pathways are activated in macrophages (Denlinger et al. 1996). Among these pathways is Erk1/2 (Lewis et al. 1998; Garrington and Johnson 1999), which is often used as a marker for LPS-induced signal transduction in different cell types (Muller et al. 1993; Sanghera et al. 1996; Schumann et al. 1998; Pyo et al. 1998; Wang et al. 2000). Several studies have suggested that Erk activation is involved in LPS-induced cellular responses, such as the addition of IL-6, TNF-α, and NO (Bhat et al. 1998; Carter et al. 1999). Park et al. (2011) found that fucoidan considerably decreased expression of TNF-α, IL-1β, iNOS, and COX-2 in LPS-induced BV2 cell through inhibiting Erk, Jnk, MAPK, and Akt pathways. The MAPK/Erk cascade is a significant signaling network associated with cellular development and function. Upon activation of cell surface receptors, cascades of intracellular phosphorylation events are initiated by Ras, Raf, and MEK, to result in the phosphorylation of Erk (Yoon and Seger 2006; Kriegsheim et al. 2009). Neuronal Erk activation is involved in a wide range of activities, including neuronal survival, proliferation, and differentiation, and is a key means of transmitting neurotrophic signals. Similarly, we found that papaverine downregulates the activation of MEK/Erk pathway and reduces the release of inflammatory factors in primary retinal microglia. Our results demonstrate mechanisms involving the activation of cAMP, PKA and MEK, Erk, and their cross-talk. Some studies have found that PI3K and PKC lead to ERK activation, which is part of the response of macrophages to inflammatory stimuli (Procyk et al. 1999). Chen et al. also found that apelin activated the expression of inflammatory cytokines in BV2 cells by PI-3K/Akt and MEK/Erk pathways (Chen et al. 2015).

The exact point at which the Erk1/2 pathway is inhibited by PKA remains to be unclear. Contrastively, PKA has been reported to activate the Erk1/2 pathway and still inhibit cell proliferation (Bornfeldt and Krebs 1999). However, studies have shown that cAMP/PKA stimulates cell proliferation by inhibiting ERK1/2, thereby facilitating activation of the PI-3K pathway (Ciullo et al. 2000). Therefore, the effects of cAMP /PKA on cell proliferation are not identical in views and the specific role of the cAMP /PKA pathway in regulating the Erk1/2 remains unclear. Several studies have discovered that adrenergic receptor (AR) regulates the activation of Erk1/2 in normal cells by activating cAMP (Enserink et al. 2002; Stork and Schmitt 2002). For example, in cardiac myocytes and bone cells, activation of PKA leads to activation of the Erk1/2 pathway (Vossler et al. 1997; Troadec et al. 2002), while in other cell types like adipocytes and endothelial cells, it appears to downregulate Erk1/2 signaling (Schmitt and Stork 2001; Schmitt and Stork 2002). cAMP/PKA regulates Erk in a variety of ways, and some studies have found that inhibitory effects of the cAMP-activated small G protein Rap1, partially reversed reverses cell proliferation and cAMP inhibition of MEK/ERK activation (Hecquet et al. 2002). Besides, some studies have showed that cAMP diminishes expression of SIRT6 by suppressing its ubiquitin-proteasome-dependent degradation, which is mediated by the Raf-MEK-Erk pathway that inhibited by PKA (Kim and Juhnn 2015). In our study, the addition of cAMP inhibitors and PKA inhibitors increased levels of phosphorylated MEK and Erk, and we demonstrated that activation of the MEK/Erk pathway may be partly regulated by cAMP/PKA. Further researches will be needed to elucidate the exact mechanisms of cross-talking between the cAMP/PKA and MEK/ERK pathways and their downstream signaling cascades that inhibit microglia activation.

## Conclusions

Our studies demonstrated that papaverine may inhibit LPS-induced activation of primary retinal microglia through the MEK/Erk pathway, and the MEK/Erk pathway may be partially regulated by cAMP/PKA.
